# Three-dimensional analysis of gait in postmenopausal women with low bone mineral density

**DOI:** 10.1186/1743-0003-11-55

**Published:** 2014-04-11

**Authors:** Abeer M ElDeeb, Amr S Khodair

**Affiliations:** 1 Department of Physical Therapy for Women's Health, Faculty of Physical Therapy, Cairo University, Cairo, Egypt; 2 Department of Obstetrics and Gynecology, Faculty of Medicine, Suez Canal University, Suez, Egypt

**Keywords:** Gait, Moment, Power, Bone mineral density, Menopause

## Abstract

**Background:**

There's lack in the literature respecting changes in the trunk and hip angles, and power profile of the lower extremities in postmenopausal women with low bone mineral density (BMD). Therefore, this study aimed to examine gait characteristics of that population, and find out which characteristics may be predictors to BMD. This may provide suitable interventions for subjects with osteoporosis.

**Methods:**

Seventeen healthy postmenopausal women and seventeen with low BMD engaged in this study. Dual X-ray Absorbiometry measured BMD at lumber (L_2–4_) and femoral neck. Qualysis gait analysis system assessed the gait pattern of each subject.

**Results:**

Compared to healthy peers, women with low BMD showed less trunk rotation (p = 0.02), hip adduction (p = 0.005) and extension moments (p = 0.008). They showed less hip power generation during early stance (H1S) (p = 0.000), and swing phase (H3S) (p = 0.005), and less hip power absorption (H2S) (p = 0.005). They also, showed less knee power absorption during terminal swing (K4S) (p = 0.002), and ankle power generation at push off (A2S) (p = 0.000). The ability of the gait variables to discriminate between subjects with or without osteopenia was (0.72%, p = 0.016) for trunk rotation, (78%, p = 0.0004) for hip adductor moment, (76%, p = 0.0013) for hip extensor moment, (87%, p < 0.0001) for H1S, (79%, p = 0.0001) for H2S, (77%, p = 0.0008) H3S, (81%, p = 0.0001) for K4S, and (93%, p < 0.0001) for A2S.

**Conclusion:**

Less power generation at the hip and ankle as well as, less power absorption at the hip and knee, may suggest that postmenopausal women with low BMD showed less propulsion, and stability during walking. Trunk rotation, hip adduction and extension moments, H1S, H2S, H3S, K4S, and A2S are significant predictors for low bone mass in the postmenopausal women.

## Introduction

Osteoporosis is a major, public, healthy problem, which increases proportionally according to age. It is a skeletal disorder compromising bone strength, and predisposing the subject to an increased risk of fractures in the hip, spine, and other sites [1].

Women after menopause are more susceptible to osteoporosis than men due to hypoestrogenism [2]. They showed changes in muscle strength and mass [3], which affect gait [4], independence, and quality of life [5].

Gait studies give a clear understanding of demands placed on the musculoskeletal and neurological systems in normal subjects and patients [6]. Few studies have explored the changes in the gait of the postmenopausal women. Women with low bone mineral density (BMD) walk with slow gait speed and large step time and stance time [7], which associated with BMD at the hip, spine, and forearm [8]. The previous studies have focused on examining the spatio-temporal parameters of gait. These parameters are only results of complicated motor pattern that do not describe the gait pattern or distinguish between gait changes or their compensations [9].

Some studies have proved that joint moments during gait affect BMD. These studies have reported significant relations between joint moments and BMD in normal subjects [10], elderly [11], and patients with hip [12] or knee osteoarthritis [13]. The previous results lead to hypothesize that gait parameters in postmenopausal women with low BMD may be different from their healthy controls. These gait parameters may be associated with BMD.

So, the first objective of this study was to compare trunk and hip kinematics, joint moments, and power profile between postmenopausal women with low BMD and healthy controls. The second objective was to find out which gait characteristics related to BMD.

## Methods

### Subjects

The database of Dual X-ray Absorbiometry (DEXA) computer, at Nasser Institute Hospital for Research and Therapy, was searched in the preceding year. Postmenopausal women with normal and low BMD were contacted, and asked to engage in this study. A doctor assessed them for inclusive and exclusive criteria of the study. They should be 50–65 years old; independent; sedentary; had a natural menopause for ≥3 years, and they did not engage in any exercise program or sport training. Women were excluded if they had a history of spondylolisthesis, diabetes, hypertension, cardiopulmonary, neuromuscular and renal diseases, thrombosis, back/leg deformities or surgeries, oophorectomy, osteoarthritis and hormonal replacement therapy.

Subjects were assigned into two groups based on WHO definitions of T-score of BMD. They were assigned to normal BMD group when T-score “at one or more skeletal sites” was within 1 standard deviations of the mean for healthy adults. Subjects, with T-score between −1.0 and −2.5 standard deviation of the mean for healthy adults, were assigned to low BMD group. Full instructions about the assessment procedures had been given to each subject, who had signed a consent form. The Ethical Committee of Faculty of Physical Therapy, Cairo University, Egypt, approved the protocol of this study.

Ten normal women and nine with low BMD completed the assessment procedures. Their results were used to decide the sample size. There was a significant difference between them in the hip extension moment, which showed a medium effect size (cohen’s d = 1.007, r = 0.45). A sample of 17 women per group should be recruited to detect a medium size effect of r = 0.45 at a power of 0.80, and alpha level of 0.05.

T-score of the normal BMD group ranged from −0.11 to −1 at L_2–4_, and −0.89 to −1 at the femoral neck. For low BMD group, it ranged from −1.23 to −2.45 at the L_2–4_, and −1.2 to −2.45 at the femoral neck. Not all postmenopausal women with low BMD received Ca and vitamin D supplements.

### Anthropometric and BMD measurements

A full history of each subject was collected at starting of this study. Weight-height scale was used to measure weight and height for all subjects. The scale was calibrated; each subject stood two times and the average of the weight was recorded. Body mass index (BMI) was calculated according to the formula; weight divided by height square (Kg/m2). DEXA (Unigramm X-ray Plus; IIb (93/42/CEE); UPG-00-174/01; Italy), measured BMD (gram/cm2) at L_2–4_ and femoral neck. Although almost subjects had DEXA scan since 6 months, BMD was measured again to gain the most accurate recent data of the subjects.

### Data collection and processing

Qualisys gait system (Qualisys Medical AB, Gothenburg, Sweden) was used to analyze the gait pattern of each subject. Qualisys provided a valid and reliable data [14,15]. It consisted of six cameras, a force plate, and a personnel computer (pc). The cameras, (type170120; 100–240 V; 50–60 HZ; 230 mA), had a capture rate of 120 frames per second. They located at suitable positions, from 10-meter walkway, to view the measurement volume. They synchronized with an AMTI^b^ kistler force plate (with external amplifier 9865) located in the middle of the walkway. The pc was installed with Q Trac and Q Gait software. The pc had a Microsoft window 98, 2nd ed., 4.10.2222 A; registered to Medical Eng. System Co.(16201-OEM- 0094512–06975); and supported by a crest computer (BE, Genuine lntel, X 86 Family 6, Model 8 stepping 6, 127.0 MBRAM).

The therapist started the gait analysis by calibrating the cameras. She placed shoulder, sacrum, and feet markers on each subject, who stood in the middle of the walkway. She adjusted Q trac at the calibrating mode; at least two cameras should pick up each marker. Then, she calibrated the measurement volume by using a wand kit (type 130440). L-shape wand was placed on the force plate with the X-axis in the walking direction. Then, T-shape wand was moved in X, Y, and Z directions to allow all cameras to pick up the markers positions in various locations. Force plate position was calibrated by placing four markers at its corners. Then, data was captured, tracked, and exported.

Twenty reflecting markers were placed on special bony landmarks of each subject according to the motion system software. They were placed as follows: the tip of each acromion, spinous process of 12^th^ thoracic vertebra, and the sacrum (between the right and left posterior superior iliac spine). Other markers were placed on the anterior superior iliac spine, greater trochanter, lateral side of the knee joint center, superior surface of the patella, tibial tuberosity, lateral malleolus, heel, and between the distal ends of the 2^nd^ and 3^rd^ metatarsals of each leg.

Then, each subject walked along the walkway three trials at her self-selected speed without knowing the presence of the force plate to step on it without targeting. The three-dimensional coordinates of the markers were calculated and filtered by Q Trac and Q Gait software. The collected data were: trunk tilt, rotation and obliquity range of motion (ROM), hip sagittal, coronal and transverse ROM, hip extension moment at midstance, adduction moment at loading response, and internal and external rotation moments. Power profile of the lower extremities in the sagittal plane was also, collected. According to Eng and Winter [16], the work done by the muscles was represented by two capital letters and one number. The first capital letter points out the joint (H = hip, K = knee, A = Ankle); the number points out the power burst position; and the last capital letter points out the plane (S = sagittal).

### Statistical analysis

Mann–Whitney *U*-test was used to compare between two groups. Spearman’s correlation coefficients assessed the relationship between BMD and gait variables. SPSS version 13.0 (SPSS Inc., Chicago, IL, USA) carried out analysis of the data. Receiver operating curve (ROC) assesses discriminative properties of the gait variables. MedCalc (version 13.0.2) calculated area under curve (AUC), sensitivity, specificity; positive predictive value (PPV) and negative predictive value (NPV). The probability value was considered significant at < 0.05.

## Results

### Subjects' characteristics

No significant difference was found between subjects’ characteristics including age, height, and postmenopausal years. However, low BMD group had lower BMI (p = 0.01) than normal group (Table 1).

**Table 1:** Subjects' characteristics

**Variable**	**Normal BMD group (N = 17)**	**Low BMD group (N = 17)**	**MannWhitney p-value**
Age (yrs)	53.23 ± 4.52	55.88 ± 4.94	0.09
Height (m)	1.57 ± 0.04	1.54 ± 0.05	0.10
BMI (Kg/m^2^)	29.91 ± 1.91	28.59 ± 1.53	0.01^*^
Postmenopausal yrs	4.94 ± 2.81	5.64 ± 2.91	0.36

Values are mean ± SD, BMD: bone mineral density, p: probability, *p < 0.05.

### Kinematics and kinetics parameters

Tables 2 and 3 represent kinematics and kinetics parameters of normal and low BMD groups. Results showed no significant differences between groups in trunk tilt, and obliquity, as well as hip angles in the sagittal, coronal, and transverse planes. Compared to normal peers, postmenopausal women with low BMD showed a decrease in the trunk rotation (p = 0.02), hip adduction (p = 0.005) and extension moments (p = 0.008). They generated less power at the hip during the early stance (H1S) (p = 0.000), and swing phase (H3S) (p = 0.005), as well as the ankle at the push off (A2S) (p = 0.000). They absorbed less power at the hip during the stance phase (H2S) (p = 0.002), and the knee during the terminal swing (K4S) (p = 0.002).

**Table 2:** Kinematics parameters of the postmenopausal women

**Variable**	**Normal BMD group (N = 17)**	**Low BMD group (N = 17)**	**MannWhitney p-value**
Trunk			
Tilt ROM	4.05 ± 1.31	4.34 ± 1.79	0.53
Rotation ROM	16.06 ± 4.46	12.60 ± 4.96	0.02^*^
Obliquity ROM	9.49 ± 2.59	8.52 ± 3.03	0.32
Hip			
Sagittal ROM	41.24 ± 7.36	38.39 ± 5.57	0.17
Coronal ROM	11.50 ± 3.68	11.42 ± 3.17	0.86
Transverse ROM	14.28 ± 2.36	12.54 ± 3.25	0.08

Values are mean ± SD, BMD: bone mineral density, p: probability, ROM: range of motion, *p < 0.05.

**Table 3:** Kinetics parameters of the postmenopausal women

**Variable**	**Normal BMD group (N = 17)**	**Low BMD group (N = 17)**	**MannWhitney p-value**
**Hip moment**			
Adduction moment (N.m/Kg)	1.01 ± 0.11	0.88 ± 0.12	0.005^**^
Extension moment (N.m/Kg)	0.49 ± 0.22	0.30 ± 0.12	0.008^**^
External rotation moment (N.m/Kg)	0.06 ± 0.04	0.06 ± 0.04	0.61
Internal rotation moment (N.m/Kg)	0.13 ± 0.04	0.12 ± 0.05	0.69
**Hip power**			
H1S (W/Kg)	0.57 ± 0.20	0.31 ± 0.10	0.000^***^
H2S (W/Kg)	0.38 ± 0.19	0.21 ± 0.10	0.002^**^
H3S (W/Kg)	0.71 ± 0.28	0.44 ± 0.19	0.005^**^
**Knee power**			
K1S (W/Kg)	0.33 ± 0.09	0.34 ± 0.13	0.71
K2S (W/Kg)	0.32 ± 0.18	0.28 ± 0.12	0.51
K3S (W/Kg)	0.67 ± 0.27	0.52 ± 0.30	0.18
K4S (W/Kg)	0.94 ± 0.37	0.53 ± 0.27	0.002^**^
**Ankle power**			
A1S (W/Kg)	0.28 ± 0.20	0.21 ± 0.10	0.31
A2S (W/Kg)	2.23 ± 0.44	1.61 ± 0.46	0.000^***^

Values are mean ± SD, BMD: bone mineral density, H: hip, K: knee, A: ankle, S: sagittal, Number: indicates number of power burst, P: probability, *p < 0.05, **p < 0.01, ***p < 0.001.

### Predictors of BMD

Table 4 shows Spearman’s correlation coefficients between BMD and gait variables. L_2–4_ BMD significantly related to the trunk rotation (r = 0.38, p = 0.02), hip adduction (r = 0.47, p = 0.005), and extension moments (r = 0.44, p = 0.009), H1S (r = 0.55, p = 0.001), H2S (r = 0.41, p = 0.017), H3S (r = 0.50, p = 0.003), K4S (r = 0.51, p = 0.003), and A2S (r = 0.66, p = 0.0001). Also, neck BMD significantly related to the trunk rotation (r = 0.36, p = 0.03), hip adduction (r = 0.43, p = 0.011) and extension moments (r = 0.46, p = 0.006), H1S (r = 0.64, p = 0.0003), H2S (r = 0.52, p = 0.004), H3S (r = 0.40, p = 0.018), K4S (r = 0.47, p = 0.004), and A2S (r = 0.65, p = 0.003).

**Table 4:** Spearman’s Correlation between BMD and gait variables

**Variable**	**L**_**2–4 **_**BMD**		**Femoral neck BMD**	
	r	p	r	p
Trunk rotation ROM	0.38	0.02^*^	0.36	(0.03)^*^
Hip adduction moment (N.m/Kg)	0.47	0.005^**^	0.43	(0.011)^*^
Hip extension moment (N.m/Kg)	0.44	0.009 ^**^	0.46	(0.006)^**^
H1S (W/Kg)	0.55	0.001^***^	0.64	(0.0003)^***^
H2S (W/Kg)	0.41	0.017 ^*^	0.52	(0.004)^**^
H3S (W/Kg)	0.50	0.003 ^**^	0.40	(0.018)^*^
K4S (W/Kg)	0.51	0.003 ^**^	0.47	(0.004)^**^
A2S (W/Kg)	0.66	0.0001 ^***^	0.65	(0.003)^**^

BMD: bone mineral density, ROM: range of motion, H: hip, K: knee, A: ankle, S: sagittal, Number: indicates number of power burst, r: Spearman’s correlation coefficient, P: probability, *p < 0.05, **p < 0.01, ***p < 0.001.

### ROC curve analysis

**Figure 1:**
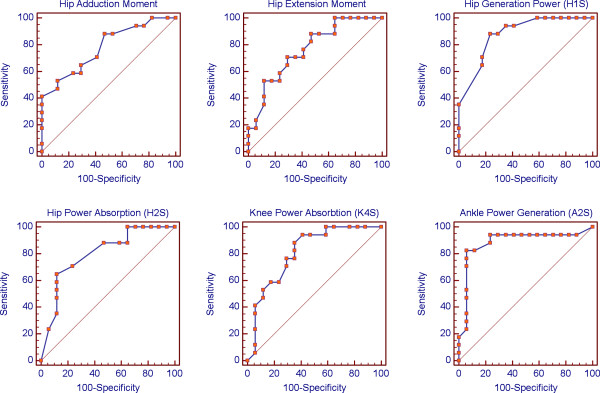
Receiver operating characteristics (ROC) curves for BMD predictors.

Figure 1 shows ROC curves of the gait variables related to BMD. Table 5 represents the discriminative properties including AUC, sensitivity, specificity, PPV and NPV. The ability of these variables to discriminate between subjects with or without osteoporosis was (72%, p = 0.016) for trunk rotation, (78%,p = 0.0004) for hip adduction moment, (76%,p = 0.0013) for hip extension moment, (87%, p < 0.0001) for H1S, (79%,p = 0.0001) for H2S, (77%, p = 0.0008) for H3S, (81%, p = 0.0001) for K4S, and (93%, p < 0.0001) for A2S. Comparison between AUC of the gait variables showed no significant differences (P > 0.05) between them in distinguishing between normal and low BMD women.

**Table 5:** ROC analysis of BMD predictors

**Variable**	**AUR (95% CI)**	**Optimal criterion**	**Sensitivity (95% CI)**	**Specificity (95% CI)**	**PPV**	**NPV**
Trunk rotation ROM	0.72 (0.54-0.86)	≤12.68	52.9 (27.8-77)	88.24 (63.2-98.5)	81.8	65.2
Adduction moment (N.m/Kg)	0.78 (0.60-0.90)	≤0.87	41.2 (18.4-67.1)	100 (80.5-100)	100	63
Extension moment (N.m/Kg)	0.76 (0.58-0.89)	≤0.34	70.6 (44–89.7)	70.6 (44–89.7)	70.6	70.6
H1S (W/Kg)	0.87 (0.72-0.96)	≤0.39	88.2 (63.6-98.5)	76.5 (50–93.2)	78.9	86.7
H2S (W/Kg)	0.79 (0.62-0.91)	≤0.23	64.7 (38.3-85.8)	88.2 (63.6-98.5)	84.6	71.4
H3S (W/Kg)	0.77 (0.60-0.90)	≤0.71	100 (80.5-100)	52.9 (27.8-77)	68	100
K4S (W/Kg)	0.81 (0.64-0.92)	≤0.82	88.2 (63.6-98.5)	64.7 (38.8-85.8)	71.4	84.6
A2S (W/Kg)	0.93 (0.72-0.99)	≤1.78	100 (66.4-100)	90 (55.5-99.7)	90	100

ROC: Receiver operating curve, AUR: area under curve, CI: confidence interval, PPV: positive predictive value, NPV: negative predictive value, H: hip, K: knee, A: ankle, S: sagittal, Number: indicates number of power burst.

## Discussion

This study aimed to provide information about changes in the gait pattern of the postmenopausal women with low BMD; and to find out which gait parameters related to bone mass. A previous study has already showed a correlation between hip joint moments produced during stepping and hip bone mass in older females [11]. However, this study did not compare between normal and low BMD group or provide information about changes occurred in the trunk, knee, and ankle joints.

In the present study, women with low BMD preserved trunk ROM in the sagittal and coronal planes and hip angles in the three-dimensional planes. However, they showed a decrease in trunk rotation. This finding was in line with Tsauo et al. [17], who reported that spinal motion performance declined, in relation to the severity of BMD, in postmenopausal women without vertebral fracture.

Postmenopausal women, with low BMD, showed a decrease in the external hip extension and adduction moments, power generation at the hip (H1S&H3S) and ankle (A2S), as well as power absorption at the hip (H2S) and knee (K4S).

Reduced external hip adduction and extension moments agreed with Hurwitz et al. [12], who reported a positive correlation between normalized femoral neck BMD and hip moments in patients with hip osteoarthritis. Reduced hip extension moment at midstance may decrease the H2S produced by eccentric contraction of the hip flexors (psoas major and illiacus) [18]. Reduced both variables may affect BMD at L_2–4_ and this finding was supported by significant positive correlations found between L_2–4_ BMD and both hip extension moment and H2S. The attachment of psoas major along the lateral surfaces of the vertebral bodies of T_12_ and L_1–5_[19] could explain the previous correlations. So, these findings suggest that reduced hip extension moment and associated eccentric hip work may reduce stress on the lumbar region and result in bone loss.

Hip power generation controls the trunk, and collapse of the stance limb; while hip power absorption decelerates the thigh extension [16]. Reduced hip power generation and absorption may represent a problem for women with low BMD. They may not be able to produce a powerful work needed for balance recovery, when large disturbance applied. Postmenopausal women, with low BMD, exhibited decreased knee absorption power produced by eccentric contraction of hamstring [18].

Muscles produce equal amounts of positive and negative work during walking. Muscles contract eccentrically to decelerate the body, and store energy for concentric contraction [16]. During this lengthening behavior, high muscles force is produced [20]. The mode of muscles contraction either concentrically or eccentrically, during rehabilitation of osteoporotic patients, may play a role in maintaining bone mass in the postmenopausal women. However, this needs further electromyography studies to support the previous hypothesis.

The ankle power generation at push-off decreased in low BMD group. Push-off phase is the period during which the muscles power generation is greatest, suggesting that phase is associated with overpowering of the gravitational load [21]. This reflects the importance of this phase in predicting BMD.

Previous studies reported a decreased ankle work in the elderly with limited activities. These studies reported that the elderly might compensate for the reduced ankle work by increasing concentric hip work at early stance [22], or increasing eccentric hip work during midstance [23]. Women with low BMD may not able to compensate for less ankle power due to decreased hip generation and absorption work. Ankle muscle weakness has been suggested as a possible cause for reduced ankle power work in elderly [21].

Decreased hip moments and power generation and absorption in low BMD group are not surprising results because of the strong relationship between muscle strength and bone mass. Muscles induce stains on the skeleton by producing bone bending moments as well as, compressive and tensile force [24]. Sarcopenia and osteoporosis are directly related conditions. Women with osteopenia or osteoporosis showed a greater loss of muscles tissues (sarcopenia) than their normal peers [25]. Sarcopenia results from age-related declines in alpha-motor neurons, sex steroid levels, physical activity [26], and inadequate intake of dietary vitamin D [27]. All of these factors lead to weakness and atrophy of the muscles, which contribute to frailty in elderly [28]. Decline in the activity of the muscles, as a result of weakness, decreases the mechanical stimulation of the muscles on the bones, and aggravates osteoporosis [29].

Work generation and absorption are good indicators to propel and control the lower limbs [16]. Reduced concentric work at the hip and ankle as well as, eccentric work at the hip and knee may suggest that postmenopausal women with low BMD exhibited less propulsion during walking. The previous findings may explain why osteopenic or osteoporotic patients have less stability, and great probability for falls during walking [30,31].

All gait parameters reduced in low BMD group significantly related to L_2–4_ and femoral neck BMD. Discriminative properties of these gait variables suggest using them as sensitive predictors for low BMD. Seeking for effective interventions, that addresses the affected gait characteristics, is important for maintaining gait mechanics in women with low BMD.

## Conclusions

Postmenopausal women, with low BMD, walk with less hip extension and adduction moments. They produce less power at the hip during early stance and ankle at push off. They absorb less power at the hip during midstance and knee during terminal swing. The previous findings suggest that they had less propulsion and less stability during walking. Hip adduction moment, hip extension moment, H1S, H2S, H3S, K4S and A2S are good markers to predict low L_2–4_, and femoral neck BMD in the postmenopausal women.

## Competing interests

The authors declare that they have no competing interests.

## Authors’ contributions

A. ElDeeb: Protocol/project development, Data collection or management, Data analysis, Manuscript writing/editing; AS. Khodair: Referral of the patients.

## Authors’ information

A. ElDeeb is a lecturer of Physical Therapy for Women's Health, Department of Physical Therapy for Women's Health, Faculty of Physical Therapy, Cairo University, Egypt.

A. Khodair is a professor of Obstetrics and Gynecology, Department of Obstetrics and Gynecology, Faculty of Medicine, Suez Canal University, Egypt.
